# Visual Loss Caused by Central Retinal Artery Occlusion After Bee Sting: A Case Report

**DOI:** 10.3389/fmed.2021.707978

**Published:** 2021-11-22

**Authors:** Zhitao Su, Zhongli Hu, Lei Wang, Yao Wang, Xiaoyun Fang, Panpan Ye

**Affiliations:** ^1^Eye Center, Second Affiliated Hospital, School of Medicine, Zhejiang University, Hangzhou, China; ^2^Department of Ophthalmology, Zhuji People's Hospital of Zhejiang Province, Zhuji, China; ^3^Department of Emergency, Second Affiliated Hospital, School of Medicine, Zhejiang University, Hangzhou, China

**Keywords:** bee sting, central retinal artery occlusion, thrombosis, venom, case report

## Abstract

A bee sting can lead to an extremely rare case of visual loss caused by central retinal artery occlusion (CRAO). In this study, we report a 66-year-old healthy woman who was referred to our Eye Center because of visual loss, which had occurred after bee sting 2 days earlier. The visual acuity was no light perception (NLP). Examination revealed left eyelid edema, conjunctiva congestion, a 6-mm fixed pupil, scattered retinal hemorrhage, and white-appearing ischemic retina with one small area of the normal-appearing retina temporal to the optic disk. Fundus fluorescein angiography revealed CRAO with one cilioretinal artery sparing. Her systemic workup revealed hypersensitivity, hypercoagulable state, myocardial damage, and hepatic damage. After topical and systemic treatments, the visual acuity was still NLP with improved systemic workup. In brief, CRAO may occur after bee sting, and visual acuity should be monitored for early diagnosis.

## Introduction

Sudden visual loss is an ophthalmologic emergency. Central retinal artery occlusion (CRAO), one of the common etiological factors, can lead to sudden visual loss (1, 2). Systemic conditions play an important role in the development of CRAO (3). Bee sting may lead to local and systemic reactions due to sensitization of the patient. A symptomatic spectrum of a bee sting ranges from mild local reaction to death (4). Inflammation caused by bee stings, such as keratopathy and endophthalmitis, has been well-reported (5–8). Recently, Pujari et al. described a patient with CRAO after bee sting; this patient was treated with systemic steroids and his systemic workup was normal at 1 week following the incidence, the exact pathogenesis of CRAO in this case is not clear (9). In this study, we present another case of visual loss caused by CRAO after bee sting, which was accompanied with hypersensitivity, hypercoagulable state, myocardial damage, and hepatic damage.

## Case Report

A 66-year-old healthy woman was in a coma for 2 h after a bumble bee had stung the left side of her face. When she woke up, she immediately reported obvious eyelid edema and blurred vision in left eye. In addition, some systemic symptoms, such as dizziness, headache, nausea and vomiting, and multiple diarrhea were also reported. The patient was treated with methylprednisolone, compound glycyrrhizin injection, and ceftriaxone sodium at a local hospital.

**Figure 1:**
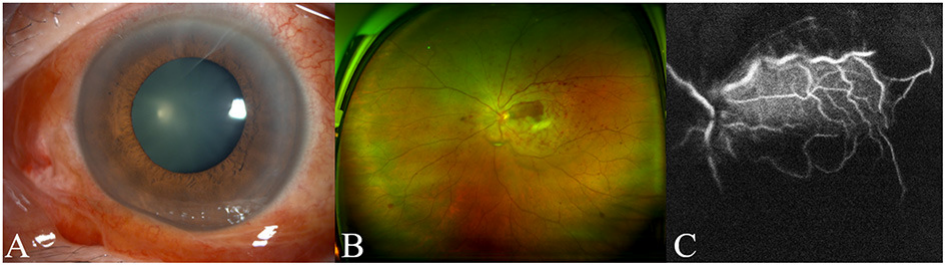
The findings of anterior segment image, fundus image, and fundus fluorescein angiography. Anterior segment photograph shows conjunctiva congestion and a 6-mm fixed pupil **(A)**. Fundus image reveals scattered retinal hemorrhage and retinal whiting with one small area of the normal-appearing retina temporal to the optic disk **(B)**. Fundus fluorescein angiography 40 s after injection of the dye showed rapid filling of one cilioretinal vessel and delayed filling of the central retinal artery **(C)**.

Forty-eight hours later, visual loss in left eye was observed by an ophthalmologist. She was referred to our Eye Center. On presentation, the visual acuity was 20/32 in right eye and no light perception (NLP) in left eye. The intraocular pressure was 16 mmHg in right eye and 19.5 mmHg in left eye. Examination of left eye revealed eyelid edema, conjunctiva congestion, a 6-mm fixed pupil (Figure 1A), scattered retinal hemorrhage, and white-appearing ischemic retina with one small area of the normal-appearing retina temporal to the optic disk (Figure 1B). The CRAO with one cilioretinal artery sparing was identified by fundus fluorescein angiography (Figure 1C). Except for mild cortical cataract, the ocular examination was unremarkable in right eye.

She was admitted to the emergency department of our hospital and systemic workup was performed. Increased total immunoglobulin E 910 IU/ml (reference 0–100 IU/ml) indicated hypersensitivity. Decreased activated partial thromboplastin time 25 s (reference 30–45 s) and increased D-dimer 590 ug/L (reference 0–500 μg/L) indicated a hypercoagulable state. Increased creatine kinase 3,340 U/L (reference 0–145 U/L) and creatine kinase-MB 68 U/L (reference 0–24 U/L), decreased left ventricular diastolic function by cardiac Doppler ultrasound and T wave change in the left anterior wall by ECG were observed, which revealed myocardial damage. Increased alanine aminotransferase 97 U/L (reference 0–34 U/L) and aspartate aminotransferase 200 U/L (reference 0–34 U/L) indicated hepatic damage. Increased lactate dehydrogenase 507 U/L (reference 140–271 U/L) may be associated with the myocardial damage and hepatic damage. Other systemic workups including blood test (routine, C reactive protein, erythrocyte sedimentation rate, electrolyte routine, procalcitonin, B-type natriuretic peptide, immunoglobulin, complement, and renal function), color Doppler ultrasound (bilateral thyroid and cervical lymph nodes, liver, gallbladder, pancreas, spleen, bilateral kidney, ureter, and bladder), cranial MRI, and high-resolution CT (orbital and chest) were within normal limits. The patient received treatments including dilation of the central retinal artery (sublingual isosorbide nitrite), reduction of intraocular pressure (intravenous mannitol), topical eye drops (Tobramycin and Dexamethasone eye drops, Pranoprofen eye drops, and Olopatadine eye drops), and systemic treatment (methylprednisolone, compound glycyrrhizin, ceftriaxone sodium, and glutathione). After 5 days of treatment, the visual acuity was still NLP in left eye with mildly decreased activated partial thromboplastin time 28.8 s (reference 30–45 s) and mildly increased D-dimer 510 ug/L (reference 0–500 ug/L), normal creatine kinase 37 U/L (reference 0–145 U/L) and creatine inase-MB 20 U/L (reference 0–24 U/L), normal aspartate aminotransferase 26 U/L (reference 0–34 U/L), mildly increased alanine aminotransferase 46 U/L (reference 0–34 U/L), and lactate dehydrogenase 351 U/L (reference 140–271 U/L).

## Discussions

Bee stings are common environmental injuries, generally resulting in some limited discomfort and swelling at the site of the sting with minimal residual effect. Occasionally they cause severe adverse reactions such as anaphylaxis, cardiovascular collapse, and death (10). Bee venom contains a complex mixture of biogenic amines, enzymes, and polypeptide toxins (11). A sudden release of highly concentrated biogenic amines, such as histamine in the venom, produces vasodilatation and increase in capillary permeability and an immunologic reaction to high molecular weight enzymes in the venom-induced type 1 hypersensitivity response mediated by immunoglobulin E (12–14). The patient was in coma for 2 h after bumble bee sting. When she woke up, left eyelid edema and blurred vision of the left eye were reported, following dizziness, headache, nausea and vomiting, multiple diarrhea, which indicated that the bee venom caused serious systemic symptoms. Ansotegui et al. suggest that immunoglobulin E testing is the basis for the diagnosis and evaluation of suspected allergic diseases (15); significantly increased total immunoglobulin E was observed in this patient, which provided evidence for the allergic reaction in this patient. As bee venom activates some inflammatory and vascular events resulting in occlusion in vessels or diseases in the electrical system of the heart, it may cause myocardial infarction or atrial fibrillation (16). Decreased activated partial thromboplastin time and increased D-dimer, which indicated hypercoagulable state, may attribute to the myocardial damage that was revealed by increased creatine kinase and creatine kinase-MB, ECG, and cardiac Doppler ultrasound.

Local reactions, such as pain, wheal, flare, edema, and swelling, are common and generally self-limiting. The lesions of the eye after bee stings may be caused by toxic and allergic reactions (17). Ocular features after bee sting include conjunctivitis, corneal infiltrates, cataract formation, acute iritis with keratic precipitates, hyphema, lens subluxation, and rarely retinal damage (12–14, 18). In this patient, blurred vision and visual loss in left eye were reported 2 and 48 h, respectively, after being stung, which were consistent with the fundus fluorescein angiography examination showing CRAO with one cilioretinal artery sparing. Recently, one study reported the first case of CRAO following multiple bee stings (9). The exact pathogenesis of CRAO in this case is not clear, because the systemic workup including blood investigations, carotid Doppler, and echocardiography was within normal range at 1 week following the incidence, which might be attributed to steroid-induced resolution of allergic and toxic reactions. A transient vasospasm of the central retinal artery *via* the Kounis reaction following bee stings appears as the most likely mechanism of CRAO. A transient hypercoagulable state caused by large-dose envenomation is another possible mechanism. Thrombotic events either due to direct toxin effect or toxic vasculitis may also be responsible for this vascular complication (8). In our case, hypersensitivity, hypercoagulable state, myocardial damage, and hepatic damage were observed 2 days after bee sting. After 5 days of systemic treatment, her systemic workup was within normal range 1 week after the bee sting.

## Conclusions

In conclusion, although the exact pathological mechanism of bee-sting-related CRAO is unclear, there is a possibility that the thrombosis, which was induced by a component of the venom at the early stage of bee sting, blocks the central retinal artery. A further study with early systemic workup may explain the pathogenesis of CRAO after bee sting. This case emphasizes that CRAO may occur after the bee sting, and clinicians should pay attention to the visual acuity in the event of eyelid swelling.

## Data Availability Statement

The original contributions presented in the study are included in the article/supplementary material, further inquiries can be directed to the corresponding author.

## Ethics Statement

The studies involving human participants were reviewed and approved by the Ethics Committee of the Second Affiliated Hospital, School of Medicine, Zhejiang University. The patients/participants provided their written informed consent to participate in this study. Written informed consent was obtained from the individual(s) for the publication of any potentially identifiable images or data included in this article.

## Author Contributions

ZS: conception, design, image interpretation, and manuscript preparation. ZH: data collection and analysis and manuscript preparation. LW: systemic examination collection and interpretation. YW: image interpretation and diagnosis. XF: patient treatment and data collection. PY: conception and design. All authors read and approved the final manuscript.

## Funding

This study was supported by the Natural Science Foundation of Zhejiang Province (LY15H120001).

## Conflict of Interest

The authors declare that the research was conducted in the absence of any commercial or financial relationships that could be construed as a potential conflict of interest.

## Publisher's Note

All claims expressed in this article are solely those of the authors and do not necessarily represent those of their affiliated organizations, or those of the publisher, the editors and the reviewers. Any product that may be evaluated in this article, or claim that may be made by its manufacturer, is not guaranteed or endorsed by the publisher.
